# Supplementary material for: The Systems Biology Research Tool: evolvable open-source software
Source: BMC Syst Biol. 2008 Jun 29;2:55. doi: 10.1186/1752-0509-2-55 (PMC2446383; doi:10.1186/1752-0509-2-55)
Supplement: Additional file 1 — SBRT Archive. An archive of the current version of the Systems Biology Research Tool. [file 1752-0509-2-55-S1.zip › sbrt-1.4.0/doc/developers_guide/api/sbrt/kernel/math/stat/comp/MannWhitneyResults.html]

MannWhitneyResults


|  |  |  |
| --- | --- | --- |
| **Overview** \| **Package** \| **Class** \| **Use** \| **Tree** \| **Deprecated** \| **Index** \| **Help** | |  |
| **PREV CLASS**   **NEXT CLASS** | **FRAMES**    **NO FRAMES**     **All Classes** |  |
| SUMMARY: NESTED \| FIELD \| CONSTR \| METHOD | DETAIL: FIELD \| CONSTR \| METHOD |  |


---


## sbrt.kernel.math.stat.comp Class MannWhitneyResults

```
java.lang.Object
  sbrt.kernel.math.stat.comp.MannWhitneyResults
```

---

``` public class MannWhitneyResults extends java.lang.Object ```

This class is used to store the results of a Mann-Whitney U test.

**Author:**
:   This class was written and documented by Jeremiah Wright while in the
    Wagner lab.

---

**Constructor Summary**

`MannWhitneyResults(double u,
double tApprox,
int n1,
int n2)`
  
          Constructs a new set of results for a Mann-Whitney U test.

`MannWhitneyResults(double u,
int n1,
int n2)`
  
          Constructs a new set of results for a Mann-Whitney U test.


| **Method Summary** | |
| --- | --- |
| `boolean` | `equals(java.lang.Object obj)`             Indicates if the provided object is equal to this results object. |
| `double` | `getApproxT()`             Returns the t value approximated from the computed U statistic. |
| `int` | `getN1()`             Returns the size of the larger sample. |
| `int` | `getN2()`             Returns the size of the smaller sample. |
| `double` | `getU()`             Returns the computed U statistic. |
| `java.lang.String` | `toString()`             Returns a string represenation of these results. |

**Methods inherited from class java.lang.Object**

`clone, finalize, getClass, hashCode, notify, notifyAll, wait, wait, wait`

**Constructor Detail**

### MannWhitneyResults

```
public MannWhitneyResults(double u,
                          double tApprox,
                          int n1,
                          int n2)
```

:   Constructs a new set of results for a Mann-Whitney U test.

    **Parameters:**: `u` - the computed U statistic.: `tApprox` - the approximated t value.: `n1` - the size of the larger sample.: `n2` - the size of the smaller sample.

---


### MannWhitneyResults

```
public MannWhitneyResults(double u,
                          int n1,
                          int n2)
```

:   Constructs a new set of results for a Mann-Whitney U test.
    The approximate t value is considered to be `Double.NaN`;

    **Parameters:**: `u` - the computed U statistic.: `n1` - the size of the larger sample.: `n2` - the size of the smaller sample.


**Method Detail**

### getU

```
public double getU()
```

:   Returns the computed U statistic.

    :   **Returns:**: the computed U statistic.

---


### getApproxT

```
public double getApproxT()
```

:   Returns the t value approximated from the computed U statistic.

    :   **Returns:**: the t value approximated from the computed U statistic.

---


### getN1

```
public int getN1()
```

:   Returns the size of the larger sample.

    :   **Returns:**: the size of the larger sample.

---


### getN2

```
public int getN2()
```

:   Returns the size of the smaller sample.

    :   **Returns:**: the size of the smaller sample.

---


### toString

```
public java.lang.String toString()
```

:   Returns a string represenation of these results.

    :   **Overrides:**: `toString` in class `java.lang.Object`
    :   **Returns:**: a string represenation of these results.

---


### equals

```
public boolean equals(java.lang.Object obj)
```

:   Indicates if the provided object is equal to this results object.

    :   **Overrides:**: `equals` in class `java.lang.Object`
    :   **Parameters:**: `obj` - the object with which to compare. **Returns:**: `true` if `obj` is an instance of `MannWhitneyResults` and its U, t value, and sample sizes are the same as this object's; `false` otherwise.


---


|  |  |  |
| --- | --- | --- |
| **Overview** \| **Package** \| **Class** \| **Use** \| **Tree** \| **Deprecated** \| **Index** \| **Help** | |  |
| **PREV CLASS**   **NEXT CLASS** | **FRAMES**    **NO FRAMES**     **All Classes** |  |
| SUMMARY: NESTED \| FIELD \| CONSTR \| METHOD | DETAIL: FIELD \| CONSTR \| METHOD |  |


---
